# Molecular Cytogenetics of* Pisum sativum* L. Grown under Spaceflight-Related Stress

**DOI:** 10.1155/2018/4549294

**Published:** 2018-12-06

**Authors:** Olga Yu. Yurkevich, Tatiana E. Samatadze, Margarita A. Levinskikh, Svyatoslav A. Zoshchuk, Olga B. Signalova, Sergei A. Surzhikov, Vladimir N. Sychev, Alexandra V. Amosova, Olga V. Muravenko

**Affiliations:** ^1^Engelhardt Institute of Molecular Biology, Russian Academy of Sciences, 119991 Moscow, Russia; ^2^Institute of Biomedical Problems, Russian Academy of Sciences, 123007 Moscow, Russia

## Abstract

The ontogenesis and reproduction of plants cultivated aboard a spacecraft occur inside the unique closed ecological system wherein plants are subjected to serious abiotic stresses. For the first time, a comparative molecular cytogenetic analysis of* Pisum sativum* L. (*Fabaceae*) grown on board the RS ISS during the Expedition-14 and Expedition-16 and also plants of their succeeding (F1 and F2) generations cultivated on Earth was performed in order to reveal possible structural chromosome changes in the pea genome. The karyotypes of these plants were studied by multicolour fluorescence* in situ* hybridization (FISH) with five different repeated DNA sequences (45S rDNA, 5S rDNA, PisTR-B/1, microsatellite motifs (AG)_12_, and (GAA)_9_) as probes. A chromosome aberration was revealed in one F1 plant. Significant changes in distribution of the examined repeated DNAs in karyotypes of the “space grown” pea plants as well as in F1 and F2 plants cultivated on Earth were not observed if compared with control plants. Additional oligo-(GAA)_9_ sites were detected on chromosomes 6 and 7 in karyotypes of F1 and F2 plants. The detected changes might be related to intraspecific genomic polymorphism or plant cell adaptive responses to spaceflight-related stress factors. Our findings suggest that, despite gradual total trace contamination of the atmosphere on board the ISS associated with the extension of the space station operating life, exposure to the space environment did not induce serious chromosome reorganizations in genomes of the “space grown” pea plants and generations of these plants cultivated on Earth.

## 1. Introduction

The presence of growing plants aboard a spacecraft is important for creating and supporting a sustainable living environment during long-term space missions, and in the near future plant systems will become important components of any long-duration exploration scenario. At the same time, the ontogenesis and reproduction of plants occur inside the unique closed ecological system wherein plants undergo serious abiotic stress which can be induced by a number of factors including changes in gravity, radiations, vibration, aboard air composition with limited exchange of gases, humidity, nutrients, temperature, and light. They are often associated with reprogramming of gene expression and can influence plant growth, development, and yield [1–3]. Specifically, seed size reduction was observed in* Arabidopsis thaliana *(L.) Heynh,* Brassica rapa *L., and* Triticum aestivum* L. grown for full life cycles under microgravity conditions aboard the International Space Station (ISS) [4–7]. Changes in the cell wall metabolism were revealed in* A. thaliana* cultivated under microgravity conditions in space [8]. In* Hordeum vulgare* L.,* A. thaliana*, and* B. rapa*, overexpression of some genes associated with stress response proteins (heat shock proteins (HSP), pathogenesis-related proteins, and antioxidant proteins) was detected under space environment [9–12]. Besides, differential organ-specific proteome responses to spaceflight environment were revealed in leaves and roots from* A. thaliana* [13]. Also, chemical contamination of the artificial atmosphere aboard a spacecraft can influence the growth and development of plants cultivated there [1, 5, 14].

The “Lada” space greenhouse installed inside the Russian Segment of the International Space Station (RS ISS) provides optimal conditions for plant growth and development [15, 16]. It was shown that plants of* Pisum sativum* L. (*Fabaceae*) cultivated in the “Lada” greenhouse during four successive expeditions (Expeditions-7–Expeditions-10) maintained their reproductive functions and viable seeds for four full life cycles [17]. Analysis of genetic polymorphism by random amplified polymorphic DNA (RAPD) did not reveal changes in “space” plants compared to the ground control [17, 18]. In karyotypes of these “space” samples, significant chromosomal rearrangements were also not detected though polymorphic organization of constitutive heterochromatin (C-band polymorphism) was observed [19]. However, the results of the 15-year monitoring of volatile organic compounds in the air onboard the ISS demonstrated that, with the extension of the space station operating life, the total chemical contamination gradually built up and diversity and toxicity of the compounds increased [20]. Over time, this process can influence the ontogenesis of the plants cultivated there and/or induce changes (e.g., chromosome aberrations) in their genomes [1, 5, 14].

In eukaryotes, heterochromatin plays a key role in epigenetic regulation of gene expression. It was shown that heterochromatin gives rise to small interfering RNAs (siRNAs) derived from transposable elements or DNA repeats [21, 22]. In plants, heterochromatic siRNAs are the most abundant class of small RNAs which play important roles in gene regulation by means of RNA-directed DNA methylation [22–25]. Environmental stress factors can induce structural changes in heterochromatin [22, 26]. C-heterochromatin comprises different repeated DNA sequences including highly repeated (satellite) DNA, transposable elements and also microsatellites, or simple sequence repeats (SSRs) which consist of tandem duplications of 1–6 bp motifs [27, 28]. Microsatellites play diverse functional roles in eukaryotic genome (e.g., modulation of gene expression, regulation of chromatin organization, DNA metabolic processes, and RNA structure) [29]. Besides, SSRs are effective genetic markers due to their common length polymorphism, and they are widely used in genetic studies [30]. Microsatellites in coding sequences can be directly linked to gene function, and mutations in microsatellites may induce the functional genomic changes providing a basis for quick adaptations to environmental changes [31–33].

The rRNA genes can serve as excellent markers in phylogenetic and cytogenetic studies of plants. These genes are organized into two distinct families (i.e., 45S and 5S rDNA) that occur as tandem repeat arrays at specific chromosomal regions. Due to high copy number, detection of rDNAs is highly reproducible and provides valuable information concerning chromosomal evolution [34]. In plant genomes, the copy numbers and chromosomal distribution of rDNAs can vary rapidly even within intraspecific taxa and can therefore provide chromosomal landmarks for genome plasticity [34–36].

Thus, DNA repeated sequences play a significant role in plant genome adaptation to stress factors, and therefore it is important to investigate the karyotype polymorphism and chromosomal distribution of these DNA fractions in plants cultivated aboard a spacecraft. In the present study, molecular cytogenetic characterization of* P. sativum* plants grown on board the RS ISS during Expedition-14 and Expedition-16 (ISS-14 and ISS-16) and also plants of the succeeding generations cultivated on Earth was performed in order to reveal possible chromosome changes in the* P. sativum* genome. The karyotypes of these plants were studied by multicolour fluorescence* in situ* hybridization (FISH) with five different repeated DNA sequences (45S rDNA, 5S rDNA, PisTR-B/1, microsatellite motifs (AG)_12_, and (GAA)_9_) as probes.

## 2. Materials and Methods

### 2.1. Plant Material

Seeds of line 131 of* P. sativum *(2n=14) (generation F0) were obtained from the collection of Department of Biology of M.V. Lomonosov Moscow State University [18].

During the ISS Expedition-14 and Expedition-16, these seeds (generations F0-14 and F0-16, correspondingly) were grown in the Lada space greenhouse installed inside the RS ISS [17]. The seeds were germinated after irrigation with water, and the seedlings were cultivated in Lada at 23 ± 1°C and 40 to 50% humidity under 24 h lighting. Fluorescent bulbs of the lighting unit generate photosynthetically active radiation with the flux at the root module surface of 250 *µ*mol m^−2^ s^−1^ minimum at a distance of 150 mm away from the light emitting surface. The moisture supply to the substrate was controlled at a constant, optimal level by the Lada system using* in situ* moisture sensors. The substrate consisted of the porous ceramic soil conditioner Turface (Profile Products, Buffalo Grove, IL) fertilized with Osmocote (14N-14P-14K) (Scotts Professional, Geldermalsen, Netherlands).

During the ISS-14, the experiment was being carried out for 78 days (11/01/2007–13/04/2007) by a flight engineer M. Tyurin. During the ISS-16, the experiment was being carried out for 79 days (10/01/2008–13/04/2008) by a flight engineer Yu. Malenchenko. After completion of each experiment, the seedpods with the “space grown” seeds (F1 generations (F1-14 and F1-16)) were harvested and transported back to Earth for further analyses.

The “space grown” seeds were stored until use under aseptic conditions at 3-4°C and a relative humidity of 13-14 % which enabled successful long-term seed storage [37, 38].

Then “space grown” seeds were germinated and used for chromosome spread preparation and also further postflight planting (F2 generations (F2-14 and F2-16)). The seeds of line 131 cultivated only on Earth were used as a control. Ground control and postflight cultivation were carried out using the same substrate and similar greenhouse and at the same conditions.

### 2.2. Chromosome Slide Preparation

For FISH, the modified technique of chromosome spread preparation from pea root tips was applied [39]. The seeds were germinated in Petri dishes on the moist filter paper at room temperature. Root tips (of 0.5 cm) were excised and treated overnight (16-20 h) in ice-cold water. After the pretreatment, the root tips were fixed in ethanol:acetic acid (3:1) for 3–24 h at room temperature. Before squashing, the roots were transferred into 1% acetocarmine solution in 45% acetic acid for 15 min. The cover slips were removed after freezing in liquid nitrogen. The slides were dehydrated in 96% ethanol and then air-dried.

### 2.3. DNA Probe Preparation

The following probes were used for FISH:

(1) pTa71: a 9-kb-long sequence of common wheat encoding 18S, 5.8S, and 26S rRNA genes including spacers [40]. This DNA probe was labelled directly with SpectrumAqua and SpectrumRed fluorochromes (Abbott Molecular, Wiesbaden, Germany) by nick translation according to manufacturers' protocols.

(2) pTa794: a 420-bp-long sequence of wheat containing the 5S rRNA gene and intergenic spacer [41]. This DNA probe was labelled directly with SpectrumRed fluorochrome (Abbott Molecular, Wiesbaden, Germany) by nick translation according to manufacturers' protocols.

(3) The oligo-(GAA)_9_ probe labelled with fluorescein-12-dUTP (Roche Diagnostics, Mannheim, Germany) and oligo-(AG)_12_ probe labelled with Cy3-dUTP (DNA Synthesis, Moscow, RF). These probes were synthesized using a synthesizer ABI 394 (Applied BioSystems, Redwood City, USA) in the laboratory of biological microchips of Engelhardt Institute of Molecular Biology of RAS, Moscow, RF.

(4) The PisTR-B/1 repeat sequence (GenBank number AF300830.1) (Invitrogen, California, USA) used for identification of* P. sativum* chromosomes [42]. This DNA probe was labelled directly with fluorochromes Platinum*Bright* 415 and Platinum*Bright* 495 by Nucleic Acids Labeling Kits (Kreatech Diagnostics, Amsterdam, Netherlands) according to manufacturers' protocols.

### 2.4. FISH Procedure

Before FISH procedure, chromosome slides were pretreated with 1 mg/ml of RNase A (Roche Diagnostics, Mannheim, Germany) in 2xSSC at 37°C for 1 h and then washed three times for 10 min in 2xSSC. The slides were dehydrated in a series of 70%, 85%, and 96% ethanol solutions and then air-dried. The hybridisation mixture (22 *μ*l) containing 40 ng of each labelled probe was added to each slide. Coverslips were placed on the slides and sealed with rubber cement. Slides with DNA probes were codenatured at 74°С for 5 min, placed in a moisture chamber, and hybridised overnight at 37°C. After removing the coverslips, the slides were washed with 0.1xSSC at 44°C for 8 min and with 2xSSC at 44°C for 8 min with the final 5 min wash in 2xSSC at room temperature. For oligo-(GAA)_9_ probe labelled with fluorescein-12-dUTP, fluorescent signal amplification using FITC-Alexa 488 antibodies (VectorLabs, Youngstown, USA) was performed. After incubation for 60 min at 37°C with the detection mixture, the slides were washed two times with 2xSSC for 5 min and once in 1xPBS for 5 min each at room temperature. The slides were dehydrated and air-dried in the dark. After FISH procedure, the slides were stained with 0.125 *μ*g/ml DAPI (Serva, Heidelberg, Germany) dissolved in Citifluor anti-fade solution (UKC Chem. Lab., Canterbury, UK).

### 2.5. Chromosome Analysis

For identification of pea chromosomes, we used the molecular cytogenetic marker PisTR-B/1, developed earlier for cytogenetic classification of* P. sativum* in which the chromosome numbering was brought in line with the genetic (linkage group) identification [43, 44]. For investigation of possible chromosomal polymorphism, the oligonucleotide (AG)_12_ and (GAA)_9_ probes were used for F1 and F2 plants (ISS-14 and 16). At least 8-10 individual plants from each specimen (control, F1, and F2 plants (ISS-14 and ISS-16)) were used for the karyotype analyses. At least 15 metaphase plates from each individual were analyzed. The slides were examined using an Olympus BX-61 epifluorescence microscope (Olympus, Tokyo, Japan). Images were captured with monochrome charge-coupled device camera (Cool Snap, Roper Scientific, Inc., Sarasota, FL, USA). Then they were processed with Adobe Photoshop 10.0 software (Adobe, Birmingham, USA).

## 3. Results

### 3.1. Plant Vegetation Period and External Features of the Plants

**Figure 1:**
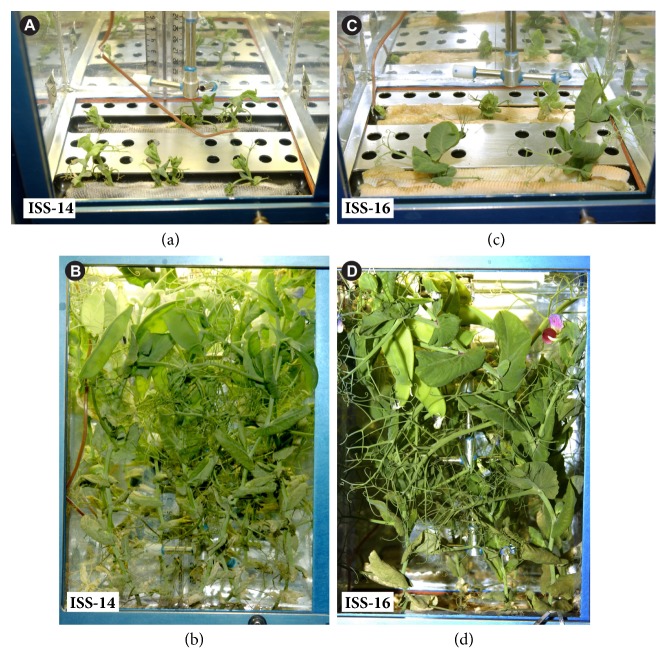
***P. sativum* plants in the Lada greenhouse aboard the RS ISS.** Initial vegetation stages of pea plants were longer during the ISS-14 (a) compared to the ISS-16 (c). Adult plants came into flower and formed seedpods during both the ISS-14 (b) and ISS-16 (d).

The experiment carried out during the ISS-14 showed that the plant vegetation period was about 80 days which was 10-12 days longer than it had been observed in the control ground experiments. This period of the seed-to-seed cycle was found to increase due to slower initial vegetation stages. The experiment carried out during ISS-16 showed that the plant vegetation period was about 65-70 days which was roughly comparable with the results of the control ground experiments. External features of the plants did not differ essentially from the control specimens (Figure 1).

### 3.2. Chromosomal Markers Revealed by FISH

For the first time, a comparative molecular cytogenetic analysis of pea plants grown on board the RS ISS during the Expedition-14 and Expedition-16 and also their succeeding (F1 and F2) generations cultivated on Earth was performed using multicolour FISH with five different repetitive DNAs (45S rDNA, 5S rDNA, the PisTR-B/1 repeat, microsatellite motifs (AG)_12_, and (GAA)_9_).

**Figure 2:**
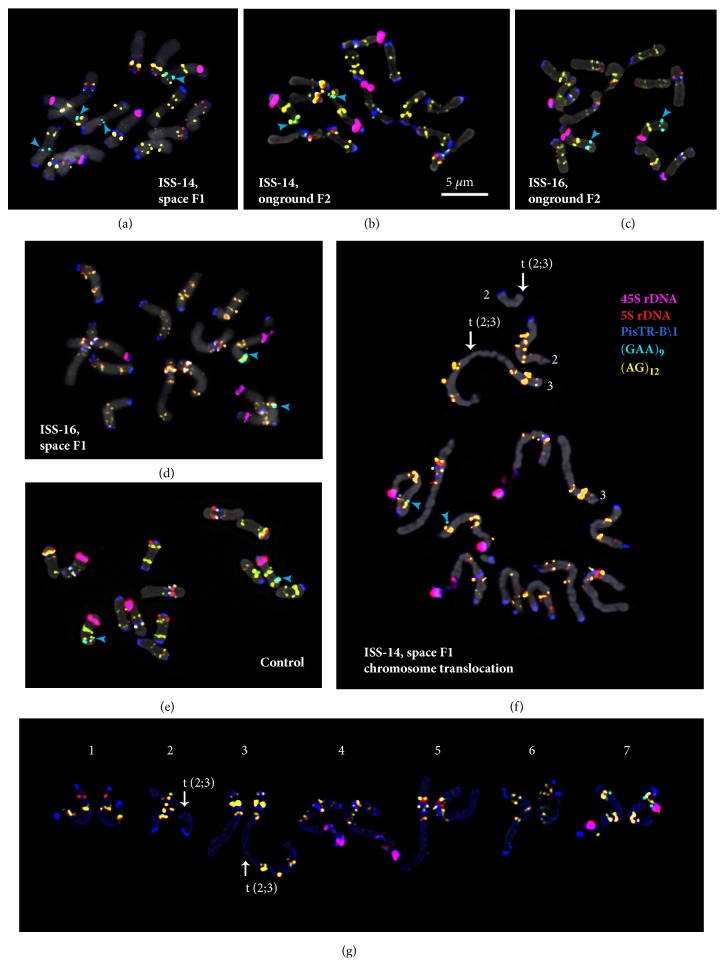
**FISH-mapping of five different DNA repeated sequences on chromosomes of experimental and control* P. sativum* samples.** Metaphase spreads of the F1-14 plant (a), F2-14 plant (b), F2-16 plant (c), F1-16 plant (d), control plant (e), F1-14 plant bearing translocation t(2;3) (f), and its karyogram (g) after FISH with 45S rDNA, 5S rDNA, PisTR-B/1, (AG)_12_, and (GAA)_9_. Arrows point to the chromosomal translocation. Heads of arrows point to (GAA)_9_ sites on chromosomes 6 and 7. The correspondent probes and their pseudocolours are specified on the right. Bar: 5 *μ*m.

**Figure 3:**
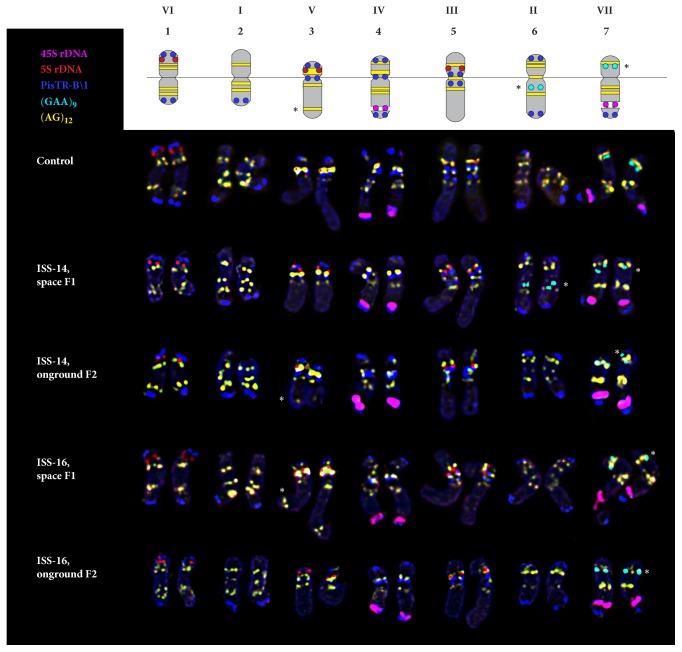
**Generalized idiogram of* P. sativum* chromosomes showing the chromosomal distribution of the examined markers and karyograms of the control and experimental samples**. Chromosome numbers and linkage groups are denoted with Arabic and Roman numerals, correspondingly. Asterisks indicate polymorphic (GAA)_9_ and (AG)_12_ sites. The correspondent probes and their pseudocolours are specified on the left.

In karyotypes of all studied* P. sativum* specimens, FISH procedure revealed 45S rDNA sites in the distal regions of the long arms of the satellite chromosome pairs 4 and 7. These chromosomes pairs can easily be distinguished as a larger satellite and a larger site of (GAA)_9_ was revealed on chromosome 7 than on chromosome 4 (Figures 2 and 3).

Hybridization sites of 5S rDNA were detected in three chromosome pairs: in the subtelomere region of the short arm of chromosome 1, in the subtelomere region of the short arm of chromosome 3 (colocalized with a large site of the oligo-(AG)_12_ probe), and also in the median region of the short arm of chromosome 5 (Figures 2 and 3).

The PisTR-B/1 repeat was mostly localized in subtelomeric regions of chromosomes (except for chromosome 5) and also in pericentromeric regions of chromosomes 3, 4, and 5.

In karyotypes of all studied* P. sativum* specimens, hybridization sites of (AG)_12_ were clustered in the intercalary regions of both arms of all chromosome pairs. Besides, a polymorphic (AG)_12_ site was revealed in the distal region of the long arm of chromosome 3 (Figure 3).

**Figure 4:**
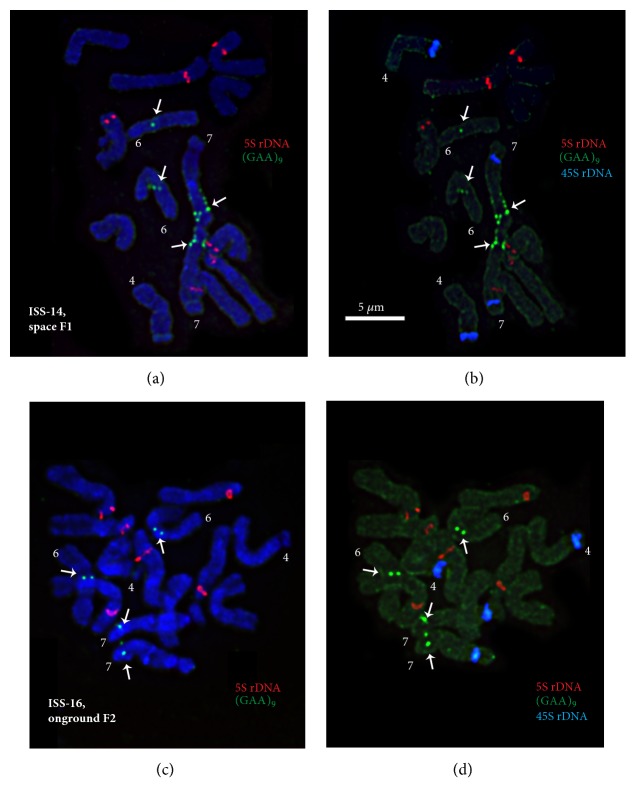
**FISH-based localization of 45S rDNA, 5S rDNA, and (GAA)**_9_ **on the metaphase spreads of F1 (ISS-14) and F2 (ISS-16)* P. sativum* plants**. Metaphase spread of a F1 pea plant (ISS-14) after FISH with 5S rDNA, (GAA)_9_ (a) and 45S rDNA, 5S rDNA, (GAA)_9_ (b). Metaphase spread of a F2 pea plant (ISS-16) after FISH with 5S rDNA, (GAA)_9_ (c) and 45S rDNA, 5S rDNA, (GAA)_9_ (d). Arrows point to the polymorphic (GAA)_9_ sites on chromosomes 6 and 7. The correspondent probes and their pseudocolours are specified on the right. Bar: 5 *μ*m.

In nearly 50% of the studied F1-14 plants, multiple hybridization signals of the oligo-(GAA)_9_ probe were detected in the short arm of satellite chromosome 7 while only one (GAA)_9_ site was observed in karyotypes of F2-14, F1-16, and F2-16 plants and also control specimens (Figure 4). In some F1 and F2 plants (ISS-14 and 16), (GAA)_9_ signals were also detected on chromosome 6 (Figures 3 and 4) which were absent in the control specimens.

Based on distribution patterns of the examined molecular cytogenetic markers, all chromosomes in the studied* P. sativum* plants were precisely identified (Figure 2). The karyograms of the control and experimental plant specimens (F1 and F2 generations) as well as the generalized idiogram showing the chromosomal distribution of the examined markers were constructed (Figure 3).

In the studied* P. sativum *plants, significant chromosomal aberrations were not revealed with the exception of one F1-14 plant bearing a reciprocal translocation between chromosomes 2 and 3 (Figure 2).

## 4. Discussion

*P. sativum *is a valuable nutritive crop having considerable natural genetic diversity though the pea evolution from its wild ancestor to the cultivated species involved selection for thousands of years [45].* P. sativum* is a genetically well-characterized species which has long been used as a classic model in genetic studies [45].

The pea karyotype has a relatively low chromosome number (2n=14) and the chromosome lengths range from of 3 to 6 *µ*m [46, 47]. The genome of* P. sativum *is rather large (1C = 4.45 Gbp) [47], and repetitive sequences make up 50-60 % of the total DNA [45, 48]. Repetitive DNA is an important component of the plant genome that is involved in genome reorganization during evolution and adaptation [34, 49]. It was shown that environmental stress factors could be responsible for quantitative changes in plant DNA (particularly, changes in satellite DNAs) [35]. Dynamic changes in the heterochromatin structure which occur during plant adaptation to a new environment can result in amplification of transposable elements as well as recombination events between repeats within satellite DNAs [50]. For instance, in* Arabidopsis *plants subjected to long-term heat stress, the increased level of transcription of satellite DNAs was observed [51]. These processes can induce structural genomic changes and formation of new structural domains and regulatory units [50]. Spaceflight-related stress factors can induce different genetic disorders in plants. For example, it was shown that space radiation resulted in various chromosomal aberrations and mitotic abnormalities in plants [52, 53]. In this study, the presence of a chromosomal aberration in one F1-14 plant can be related to peculiarities of seed formation occurring on the mother plant under space environment which were described earlier [4, 6, 7]. Alternatively, it might be a result of long-term seed storage that could induce chromosomal aberrations [37, 54].

It was reported earlier that number of mono-, di-, tri-, tetra-, penta-, and hexa-nucleotide repeat motifs in genomes of different plant species could be species-specific [55]. A number of microsatellites (ААТ)n, (AT)n, (GAA)n, and (AG)n were found to be frequent in the pea genome [48]. In the present study, the use of a combination of two microsatellites ((GAA)_9_ and (AG)_12_) with a pea specific PisTR-B as well as 5S and 45S rDNA probes allowed us to identify all the pea chromosomes as well as perform the chromosome comparison of the plants grown from the “space grown” seeds with the plants of ground control. In the* P. sativum* plants examined here, chromosomal distribution of 45S rDNA, 5S rDNA, and PisTR-B/1 mainly corresponded to the patterns reported earlier [42, 45, 56] with the exception of the only report of four chromosome pairs bearing 5S rDNA sites in the karyotype of one* P. sativum* variety [57] which is probably due to the intraspecific variability and/or particularity of that variety. Precise chromosome identification allowed us to specify the localization of the (GAA)_9_ and (AG)_12_ sites on* P. sativum* chromosomes.

In all pea specimens studied here (including the ground control), we observed a low level of polymorphism in chromosome distribution of (AG)_12_ and PisTR-B/1. However, we revealed more sites of the oligo-(AG)_12_ probe in karyotypes of the studied of* P. sativum* than it was reported earlier [57]. This might indicate the presence of the intraspecific variability in chromosome distribution of the (AG)_n_ motif in* P. sativum*. Moreover, in karyotypes of F1-14 plants, we observed multiple hybridization signals of (GAA)_9_ on satellite chromosome 7 which were absent in the control plants. Besides, in karyotypes of some F1 and F2 plants (ISS-14 and ISS-16) additional (GAA)_9_ sites were also detected on chromosome 6. Considering the fact that intraspecific polymorphism in chromosomal distribution of AG and GAA motifs was described earlier for other plants (e.g.,* Hordeum marinum* and* Brassica rapa *ssp.* chinensis*) [58, 59], the detected changes might also be an example of intraspecific genomic polymorphism. Alternatively, they can be related to plant cell adaptive responses to spaceflight-related stress factors. Microsatellites are shown to be involved in the processes of fast adaptation of plant populations to environmental changes and also phenotypic plasticity within and between generations, and gene-associated tandem repeats provide diverse variations promoting rapid development of new forms [31]. Some microsatellites act as cis-regulatory elements which can be recognized by transcription factors. For instance, it was shown that (GA)n and (GAA)n repeats were included in promoters of several plant genes and participated in regulation of processes of transcription and translation [60–63].

Plant adaptation to spaceflight conditions is known to correlate with activation of genetic and epigenetic antistress mechanisms. Large-scale analyses of transcriptome in several plant species (*B. rapa*,* H. vulgare*) showed that spaceflight-related stress factors led to oxidative stress in plants and caused an increase in expression of the genes related to abiotic and biotic stresses and also in production and activities of antioxidant enzymes [9, 10, 12]. Operating experience of hermetically sealed objects showed that artificial atmosphere is known to be a multicomponent mixture involving harmful micro impurities related to various structural classes [20, 64], and an increased level of ethylene is considered to be a major cause of oxidative stress in plants [1, 5, 14]. For example, phytotoxic influence of ethylene (1.1-2.0 mg/m^3^), which was early detected in the air aboard the Space Station Mir, resulted in changes in the productivity and morphometric characteristics of dwarf wheat cultivated there [5]. Spaceflight-related stress factors might induce plant cell adaptive responses manifested at the genomic level as changes in a copy number of microsatellite motifs. In support of this view, changes in plant vegetation period were observed during the ISS-14. Unfortunately, the other karyotypic studies of plants grown from the “space” seeds are next to none. This issue needs further investigations as the “Lada” space greenhouse is too small in size to produce a great number of seeds.

## 5. Conclusions

Significant changes in distribution of the examined repeated DNAs in karyotypes of the “space grown”* P. sativum* plants as well as in F1 and F2 pea plants cultivated on Earth were not observed if compared with control plants. The revealed changes in chromosomal distribution of the oligo-(GAA)_9_ probe in karyotypes of F1 and F2 plants might be an example of intraspecific genomic polymorphism or related to plant cell adaptive responses to spaceflight-related stress factors.

Our findings suggest that, despite gradual total trace contamination of the atmosphere onboard the ISS associated with the extension of the space station operating life, exposure to the long-term spaceflight-related stress factors did not induce serious chromosome reorganizations in genomes of the “space grown” pea plants and generations of these plants cultivated on Earth.

## Data Availability

All data used to support the findings of this study are included within the article.
